# Bacterial diversity in semen from stallions in three European countries evaluated by 16S sequencing

**DOI:** 10.1007/s11259-024-10321-3

**Published:** 2024-02-02

**Authors:** Pongpreecha Malaluang, Adnan Niazi, Yongzhi Guo, Christina Nagel, Tiago Guimaraes, Antonio Rocha, Christine Aurich, Jane M. Morrell

**Affiliations:** 1 Department of Clinical Sciences, Swedish University of Agricultural Sciences (SLU), Uppsala, 75007 Sweden; 2 Faculty of Veterinary Sciences, Mahasarakham University, Maha Sarakham, 40000 Thailand; 3 SLU-Global Bioinformatics Centre, Department of Animal Breeding and Genetics, Swedish University of Agricultural Sciences (SLU), Uppsala, SE-750 07 Sweden; 4 Science for Life Laboratory, National Bioinformatics Infrastructure Sweden (NBIS), Uppsala University, Uppsala, SE-752 36 Sweden; 5 Graf Lehndorff Institute for Equine Science, University of Veterinary Medicine, Vienna, Austria; 6 School of Medicine and Biomedical Sciences (ICBAS), University of Porto (UP), Porto, Portugal; 7 Center for the Study of Animal Sciences (CECA), ICETA, University of Porto, Campus Agrário de Vairão, Vairão, Portugal; 8 Artificial Insemination and Embryo Transfer, Department for Small Animals and Horses, University of Veterinary Medicine, Vienna, Austria

**Keywords:** Stallion seminal microbiome, Climatifc effect on bacteria, 16S rRNA sequencing, Stud hysbandry practices, Antimicroibial resistance

## Abstract

The microbiome plays a significant role in shaping the health and functioning of the systems it inhabits. The seminal microbiome of stallions has implications for the health of the reproductive tract, sperm quality during preservation and antibiotic use in semen extenders. Diverse bacteria are present on the external genital tract and a mix of commensal microorganisms populates various parts of the reproductive tract, influencing the seminal bacterial content. Other sources of bacteria include the environment, semen collection equipment, and personnel. The bacterial load can adversely affect sperm quality and fertility, particularly in artificial insemination, where semen is extended and stored before use. Antibiotics are frequently used to inhibit bacterial growth, but their effectiveness varies depending on the bacterial strains present. The aim of this study was to assess the bacterial diversity in semen from 37 healthy stallions across three European nations (Germany, Portugal, and Sweden) using 16S sequencing. Semen samples were collected from individual stallions at three AI centers; DNA extraction, sequencing, and bioinformatic analysis were performed. Differences in bacterial diversity among the stallions were seen; although bacterial phyla were shared across the regions, differences were observed at the genus level. Climate, husbandry practices, and individual variability likely contribute to these differences. These findings underscore the importance of tailoring antibiotic strategies for semen preservation based on regional bacterial profiles. The study presents a comprehensive approach to understanding the intricacies of the stallion seminal microbiome and its potential implications for reproductive technologies and animal health.

## Introduction

The microbiome has a marked influence on the reproductive system (Samper 2008; Ortega-Ferrusola et al. 2009; Guimarães et al. 2015; Varela et al. 2018; Al-Kass et al. 2019). This is apparent for the stallion reproductive tract, where the seminal microbiome has considerable implications for the reproductive health of mares bred by the stallions, for retention of sperm quality during semen storage for artificial insemination, and last but not least, for antibiotic usage in semen extenders. Previous studies have examined some of these aspects, ranging from the presence of pathogenic bacteria (Samper 2008), to the impact of bacteria on reproductive technologies (Ortega-Ferrusola et al. 2009; Varela et al. 2018). Other studies described interventions reducing bacterial load (Guimarães et al. 2015; Al-Kass et al. 2019), qualitative and quantitative analysis for bacterial contaminants (Corona and Cherchi 2009); and investigation of the microbial flora in healthy individuals (Rota et al. 2011; Pasing et al. 2013).

Numerous bacteria are constituents of the normal flora on the external genital tract of the stallion, such as *Streptococcus dysgalactiae* ssp. *equisimilis*, *Bacillus* spp., *Staphylococcus aureus*, *Escherichia coli*, *Streptococcus equi* ssp. *zooepidemicus*, *Pseudomonas* spp., and *Klebsiella* spp. (Samper and Tibary 2006). A mixture of commensal bacteria inhabits the surface of the urethral fossa, penis, prepuce, and the urethra of healthy stallions, and are transferred to the semen. Contamination of semen with bacteria from the external genitalia is difficult to avoid when collecting semen with an artificial vagina (Ortega-Ferrusola et al. 2009; Rota et al. 2011). The bacteria present in semen after collection may also originate from the environment (Pickett et al. 1999).

Bacteria in semen can have a deleterious impact on the quality of conserved sperm, affecting their viability and fertility (Aurich and Spergser 2007; Ortega-Ferrusola et al. 2009; Ramires Neto et al. 2015) This is particularly relevant for artificial insemination (AI), where the semen is extended, cooled, and stored for several hours before insemination. Bacterial growth is supported by the nutrients in the semen extender, and bacteria continue to divide at temperatures over 15 °C (Nedwell 1999).

Since bacteria in semen can cause fertility disorders in inseminated mares or may have harmful consequences for semen quality during storage, antibiotics are usually added to inhibit bacterial growth, and the temperature of the semen is reduced. However, the antibiotics added may not inhibit the contaminating bacteria if the incorrect concentration is used or if the microorganisms concerned are not sensitive to their effects (Guimarães et al. 2015). Thus, an understanding of which bacteria are present in stallion semen is necessary to determine the relevant antibiotics to include in semen extenders.

Previous studies on the bacteria present in the semen of stallions without fertility problems were made in Austria (Al-Kass et al. 2019), Germany (Pasing et al. 2013), Italy (Corona and Cherchi 2009; Rota et al. 2011), Portugal (Guimarães et al. 2015), Sweden (Al-Kass et al. 2020) and Spain (Ortega-Ferrusola et al. 2009; Varela et al. 2018; Quiñones-Pérez et al. 2021). The prevalence of the bacteria described varied among studies, perhaps because different techniques were used for bacterial identification but also possibly due to extrinsic factors such as climate, geographic location, husbandry, etc.

Traditionally, bacterial identification is culture-based, which may tend to mask the presence of some bacteria that are difficult to culture (Moretti et al. 2009). Only a few bacteria were identified from stallion semen using culture-based techniques (Corona and Cherchi 2009; Ortega-Ferrusola et al. 2009; Rota et al. 2011; Pasing et al. 2013; Varela et al. 2018; Al-Kass et al. 2019). Recently, studies on the presence of the seminal microbiome in healthy stallions using a non-culture-based method, 16S rRNA sequencing, (Al-Kass et al. 2020; Quiñones-Pérez et al. 2021) revealed many bacterial species, including some that had not been identified previously in culture-based studies.

Research on the human microbiome in the past few years has advanced using next generation sequencing (NGS) (Kuczynski et al. 2012). This method allows the ordering of nucleotides in the DNA to be defined accurately (van der Straaten 2015). The sequencing step is initiated by focusing on genes that code for the 16S (small subunit rRNA gene) of bacteria, using this gene for bacterial taxonomic definitions (Böttger 1989). More than 90% of bacterial genera and 65–83% of bacterial species can be identified in this way (Mignard and Flandrois 2006).

The objective of the present study was to characterize the bacterial species in healthy stallion semen in three European countries using 16S rRNA sequencing, and to evaluate whether climatic factors affect the microbiome.

## Materials and methods

### Animals and semen collection

Semen samples were obtained by convenience sampling. from 37 stallions at three AI centers in Germany (18 stallions), Portugal (13 stallions), and Sweden (6 stallions), where animals were kept according to national and international regulations. The stallions were free from contagious equine metritis, equine arteritis virus and equine infection anemia virus. In all cases, the stallions were housed in individual boxes with either straw bedding or wood shavings (Portugal), straw or wood chippings (Germany), or wood shavings (Sweden), renewed daily. Access to water was ad libitum. The stallions had access to outside paddocks during the day; in Germany these were sand paddocks, in Portugal grass, and in Sweden sand and grass. The animals were kept according to the European legislation regarding equids. Semen collection with an artificial vagina is considered to be a routine husbandry practice in these countries and therefore did not require ethical permission.

The semen samples were collected during different periods, to fit in with the work-load at the studs: in Portugal from October to May, apart from one in August, in Sweden during September, and in Germany in March. Semen collection was carried out with an artificial vagina (AV) after allowing the stallion to mount a dummy mare. A sterile AV and bottle were used for the collection process, and the penis was not washed before collection at any of the studs. After the collection, the sterile graduated bottle containing the ejaculate was separated from the artificial vagina and taken to the laboratory. All semen collection procedures were performed with sterile equipment and aseptic measures to avoid semen contamination. An aliquot of 1 mL raw ejaculate was stored in liquid nitrogen before transport at − 80 °C to the Clinical Sciences Laboratory at Swedish University of Agricultural Sciences for extraction of bacterial DNA.

### Fertility

For the purposes of this study, fertility was arbitrarily categorized arbitrarily as “good” if the stallions had a pregnancy rate per season above 60%, and “low” if the pregnancy rate was below 60% (Table 1).

**Table 1:** Fertility and pregnancy rate of 37 stallions

Stallion	Country	Breed	Age (years)	Fertility	%Pregnancy rate (no. of mares)
1	Sweden	Warmblood	5	Low	30
2	Sweden	Warmblood	22	Low	57
3	Sweden	Warmblood	15	Good	93
4	Sweden	Warmblood	9	Low	57
5	Sweden	Warmblood	12	Low	20
6	Sweden	Warmblood	8	Good	70
7	Germany	Warmblood	16	Low	57 (11)
8	Germany	Haflinger	9	Good	80 (39)
9	Germany	Warmblood	6	Good	95 (13)
10	Germany	Warmblood	23	Good	87 (28)
11	Germany	Warmblood	7	Good	84 (20)
12	Germany	Warmblood	5	Low	40 (12)
13	Germany	Warmblood	12	Good	65 (25)
14	Germany	Warmblood	13	Low	46 (60)
15	Germany	Warmblood	6	Good	85 (8)
16	Germany	Warmblood	11	Good	76 (20)
17	Germany	Warmblood	3	Good	50 (6)
18	Germany	Warmblood	5	Good	100 (6)
19	Germany	Warmblood	6	Good	70 (13)
20	Germany	Warmblood	6	Low	50 (2)
21	Germany	Warmblood	6	Good	95 (17)
22	Germany	Draught horse	17	Low	57 (22)
23	Germany	Warmblood	17	Good	71 (57)
24	Germany	Warmblood	9	Low	50 (2)
25	Portugal	Lusitano	5	NA	NA
26	Portugal	Lusitano	12	NA	NA
27	Portugal	Lusitano	7	NA	NA
28	Portugal	Cross-Breed	20	NA	NA
29	Portugal	BWP	7	Good	> 60
30	Portugal	Lusitano	25	Good	> 60
31	Portugal	Lusitano	11	Good	> 60
32	Portugal	Lusitano	5	NA	NA
33	Portugal	Holsteiner	4	Good	> 60
34	Portugal	Lusitano	6	Good	> 60
35	Portugal	Lusitano	16	Good	> 60
36	Portugal	BWP	11	Low	< 60
37	Portugal	Lusitano	12	NA	NA

NA = no information available

### DNA extraction

The DNA extraction was performed on 10 µL of semen using a QIAamp DNA Mini Kit according to the manufacturer’s instructions for the simultaneous purification of genomic DNA from cells. All samples were centrifuged; the supernatant was removed, and only pelleted cells were used. The purity and concentration of the DNA were tested using a NanoDrop 8000 Spectrophotometer (Thermo Scientific, Waltham (HQ), MA, USA). The DNA purity was considered adequate when the 260/280 ratio was between 1.7 and 1.9, and the concentrations were between 3.87 and 243.83 ng/µL. The DNA samples were stored at − 80 °C until further preparation.

### 16S rRNA sequencing

A two-step amplification protocol was used to prepare the 16S region of the bacterial DNA content for Illumina sequencing. The primers and cycling protocols are presented in Table 2. The reaction volume of the first step was 21 µL containing 4 µl of sample, 10 µl KAPA HiFi HotStart ReadyMix (Roche), 0.2 µl BSA (Thermo Scientific), 2 µl Primer mix (7.5 µM solution, containing 341 F and 805R, forward and reverse primers), and 4.8 µl ultra-pure water. For the second step, the reaction volume was 20 µL containing 6 µL of the purified DNA template from the first PCR step, 10 µl KAPA HiFi HotStart ReadyMix, and 4 µl of indexing primer mix (i5 and i7 indexing primer, 2.5 µM). Both PCR set-up and bead clean-up were performed in duplicate with Agilent NGS workstation Bravo (Agilent Technologies, USA) in a 96-well plate format. As the samples varied in amounts of bacterial DNA, a test with primers for the first PCR was performed to estimate the appropriate amount for the protocol in each case. The concentrations were estimated with a Qubit 3.0 fluorometer using the High Sensitivity DNA kit. Final bead clean-up was performed after the second PCR using MagSi-NGS prep plus (Tataa). In this step, free primers were removed and the amplicon was purified by binding the DNA to magnetic beads, washing and releasing the DNA in the elution buffer (Qiagen).

**Table 2:** Primer combination and thermal cycling conditions used to quantify the 16S rRNA. To increase the diversity of the final library, each primer contains 0–7 nucleotides or 0–3 nucleotides for phasing purposes

Primer 16S rRNA	Sequences (5′-3′)	Terminal Cycling	Reference
341 F	ACACTCTTTCCCTACACGACGCTCTTCCGATCT-[N[0–7]]-CCTACGGGNGGCWGCAG	(98 °C 2 min); (98 °C 20 s, 54 °C, 20 s, 72 °C 15 s) × 20; (72 °C 2 min)	(Wu et al. 2015)
805R	GTGACTGGAGTTCAGACGTGTGCTCTTCCGATCT-[N[0–7]]-GACTACHVGGGTATCTAATCC	(98 °C 2 min); (98 °C 20 s, 55 °C 30 s, 72 °C 30 s) × 8; (72 °C 2 min)	

N[0–7] = nucleotides used for phasing

### 16S rRNA analysis

Analysis of 16S rRNA sequencing data was performed using the Nextflow pipeline ampliseq v1.1.2 (https://github.com/nf-core/ampliseq). Briefly, the quality of the sequencing data analysed using FastQC (Andrews 2010), followed by trimming of primer sequences from the reads using cutadapt v2.7 (Martin 2011). Sequencing reads were denoised, dereplicated, and filtered for chimeric sequences using DADA2 (Callahan et al. 2016). Amplicon sequence variants (ASVs) were obtained from the processed sequences and taxonomically classified from phylum to species level after clustering at 99% similarity using the SILVA v132 database (Quast et al. 2013) by applying Naive Bayes classifier (Bolyen et al. 2019). Any ASVs classified as Mitochondria or Chloroplast were removed.

### Statistical analysis

For the microbiome data, alpha and beta diversity were calculated using an R package, Phyloseq v1.44.0 (McMurdie and Holmes 2013). Alpha diversity significance for bacterial diversity and richness within the samples was determined using one-way ANOVA with a false discovery rate corrected *p*-value < 0.05. Species evenness (Pielou) significance was calculated using the Kruskal-Wallis test. Beta diversity significance was determined using overall and pairwise PERMANOVA tests with a Bonferroni corrected *p*-value < 0.05. LEfSe analysis with the effect size (LDA score) was used to compare the relative abundance between Good fertility (pregnancy rate > 60%) and low fertility stallions from Germany.

## Results

**Fig. 1:**
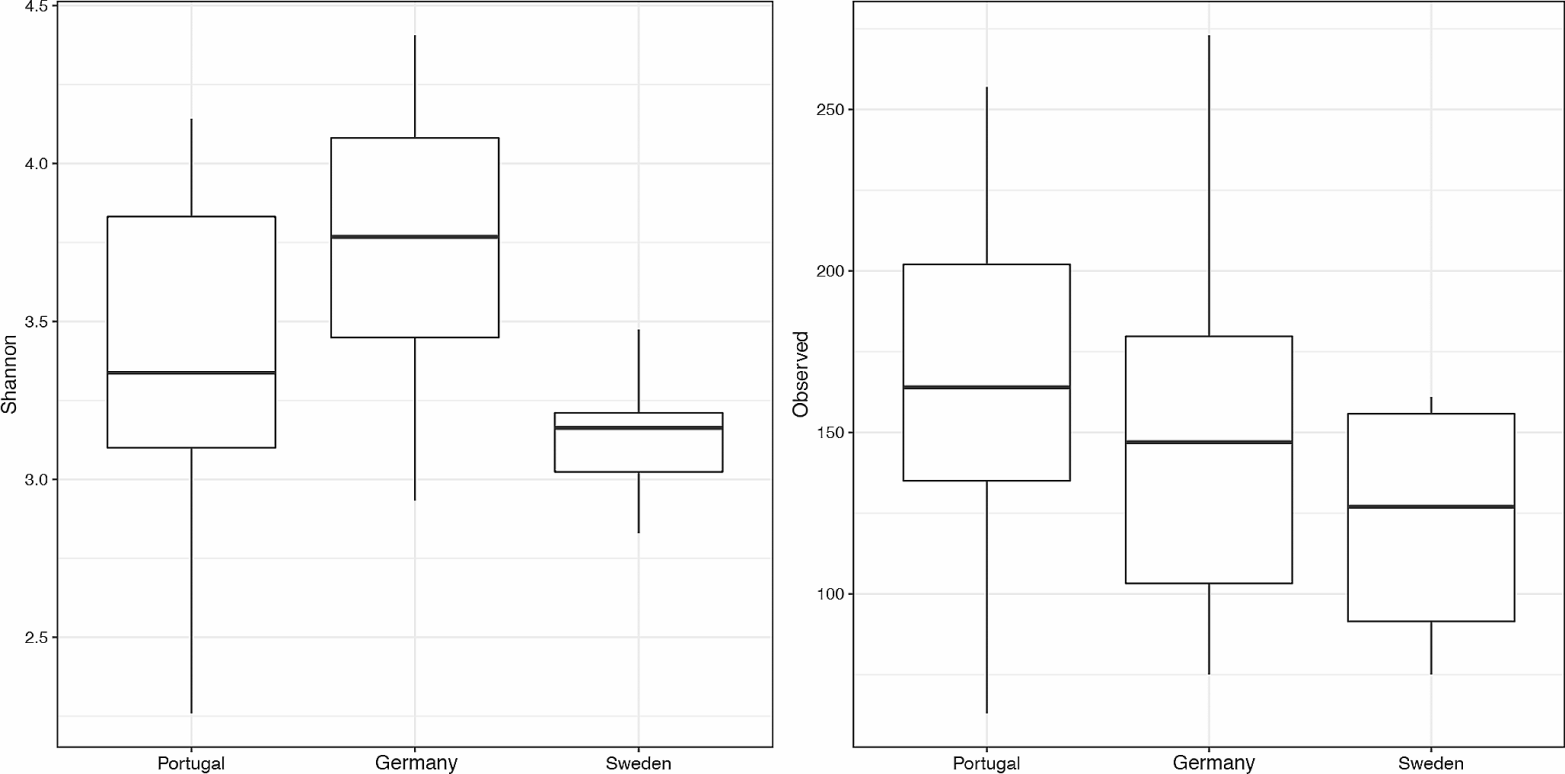
Alpha diversity measures of stallion semen samples. (Left) Distribution of Shannon alpha diversity values between countries, *p* = 0.017; (Right) Distribution of observed amplicon sequence variants (ASVs) between countries *p* > 0.05. The horizontal line in the box indicates the median

The microbiome in semen from stallions in three countries (Germany, Portugal, and Sweden) contained 1,908 identifiable ASVs. Bacterial diversity differed significantly among the different countries (Shannon Index, *p* = 0.017) (Fig. 1**-Left**). However, there was no significant difference in richness (observed ASVs, *p* > 0.05) (Fig. 1**-Right**) and no significant community evenness (Pielou, *p* > 0.05) among Germany, Portugal, and Sweden (data not shown).

**Fig. 2:**
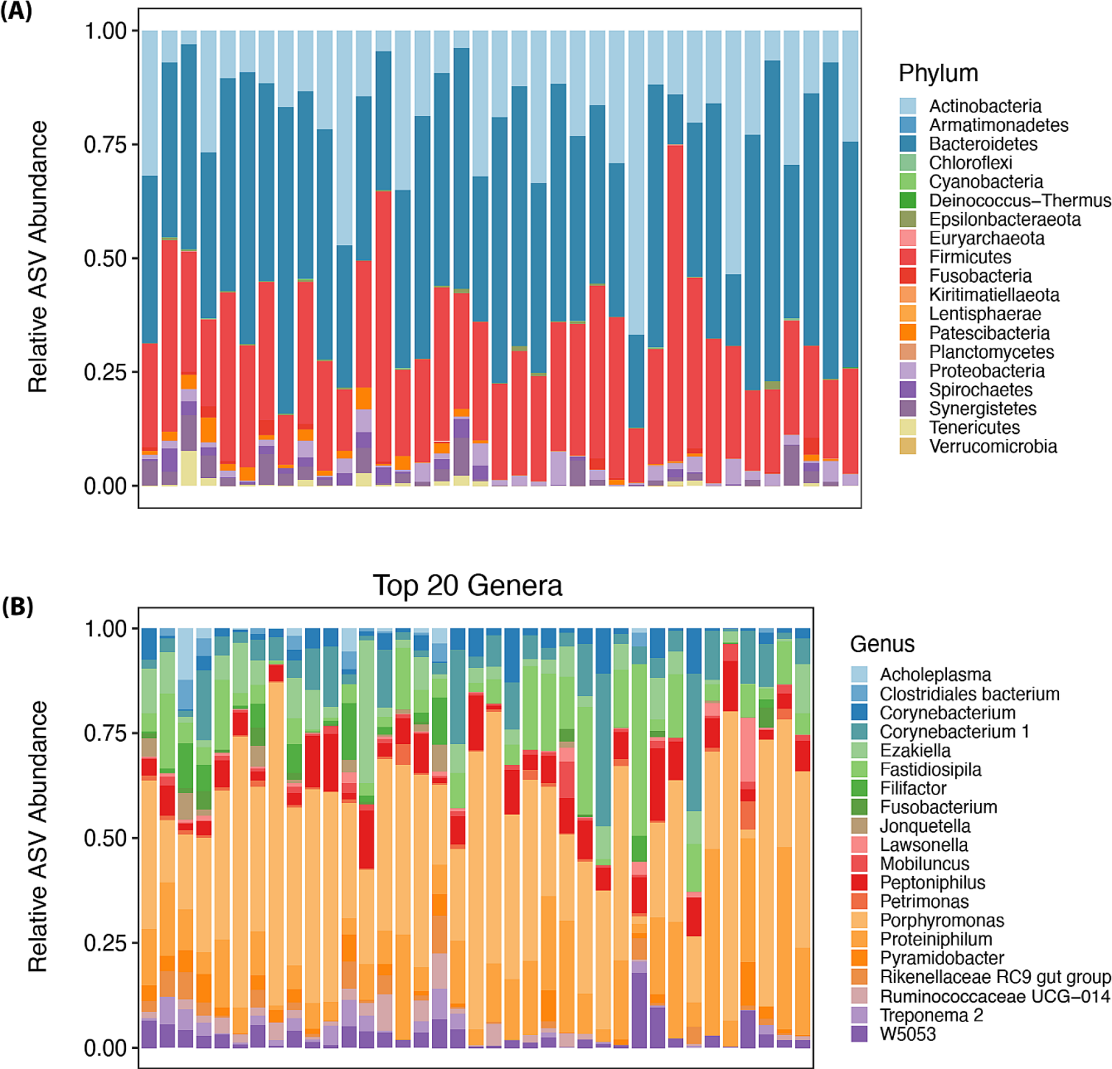
Distribution of the detected phyla (**A**) and 20 most abundant genera (**B**) in the semen of 37 stallions identified by 16S rRNA sequencing

Nineteen bacterial phyla were identified; the ten most frequently seen were Bacteriodetes, Firmicutes, Actinobacteria, Synergistetes, Proteobacteria, Spirochaetes, Patescibacteria, Tenericutes, Fusobacteria, and Epsilonbacteraeota (Fig. 2A). The occurrence of the twenty most abundant bacterial genera is shown in Fig. 2B, with the ten most observed genera being Peptoniphilus, Proteiniphilum, Fastidiosipila, Corynebacterium 1, Petrimonas, Corynebacterium, W5053, Pyramidobacter, Ezakiella, and Lawsonella (Fig. 2B).

**Fig. 3:**
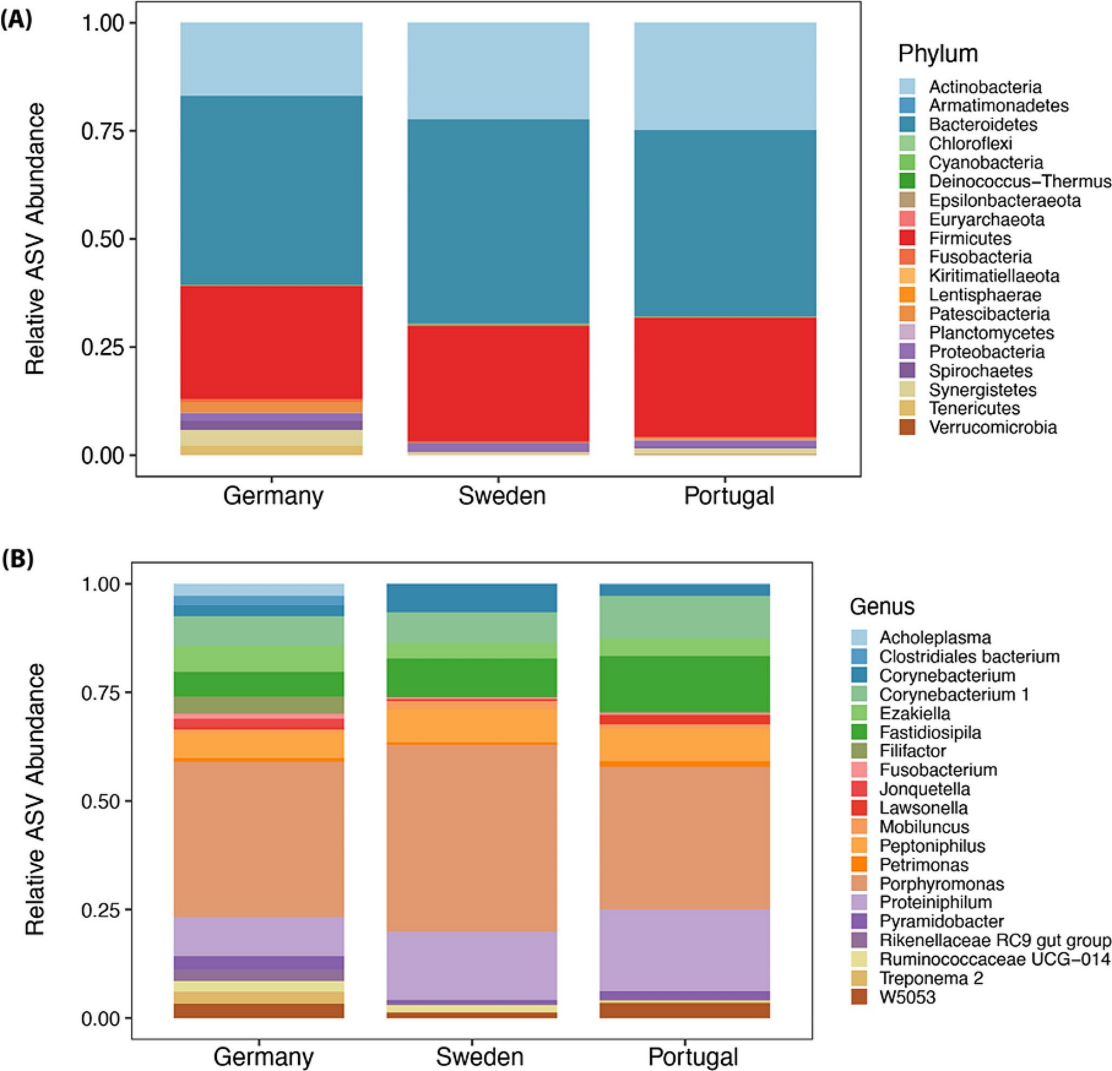
Mean relative abundance of phyla (**A**) and top 20 genera (**B**) between the countries: Germany, Sweden, and Portugal

Phyla identified in each country are shown in Fig. 3A; the twenty most abundant bacterial genera are shown in Fig. 3B. The dominant phyla in Germany, Portugal, and Sweden were Bacteriodetes, Firmicutes, and Actinobacteria, constituting about 80–95% relative ASV abundance. The three most dominant genera in Germany were *Peptoniphilus, Proteiniphilum*, and *Corynebacterium 1*, representing approximately 40% relative ASV abundance. In Portugal and Sweden, *Peptoniphilus*, *Proteiniphilum* and *Fastidiosipila* were dominant, constituting about 70% relative ASV abundance.

**Fig. 4:**
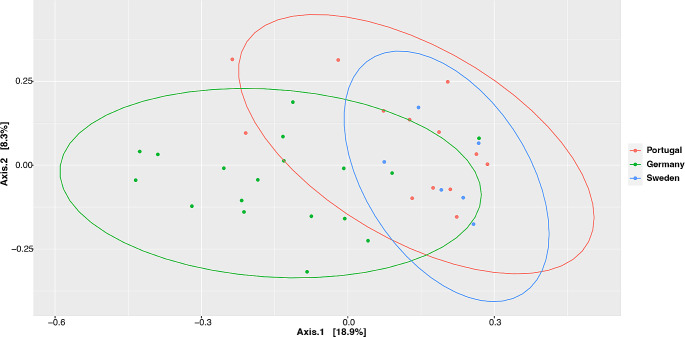
Bray-Curtis pairwise distances between countries. Axes indicate the proportion of variability explained by that axis. Each colored dot represents a sample within a country

**Fig. 5:**
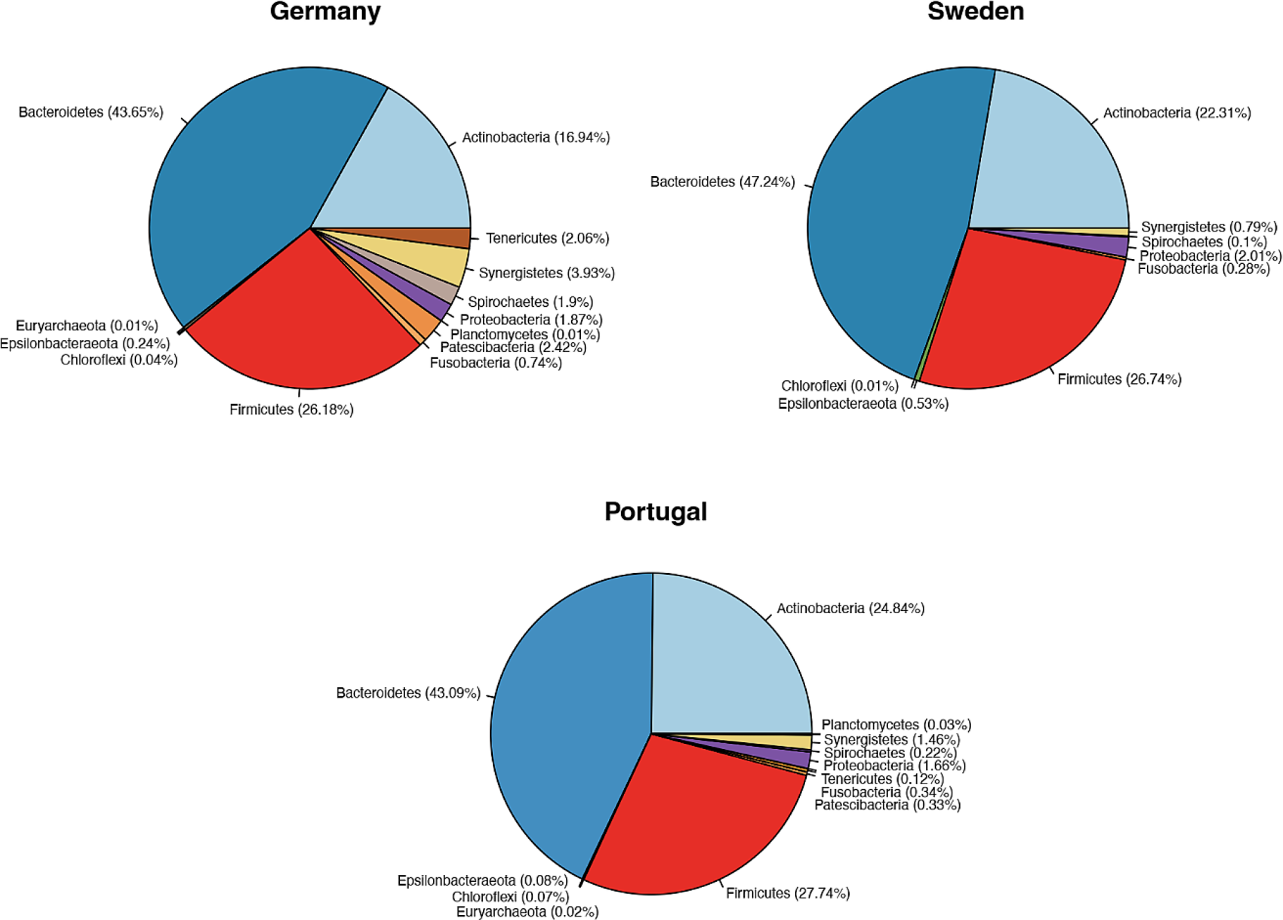
Mean relative abundance of Phyla between the countries: Germany, Sweden, and Portugal

**Fig. 6:**
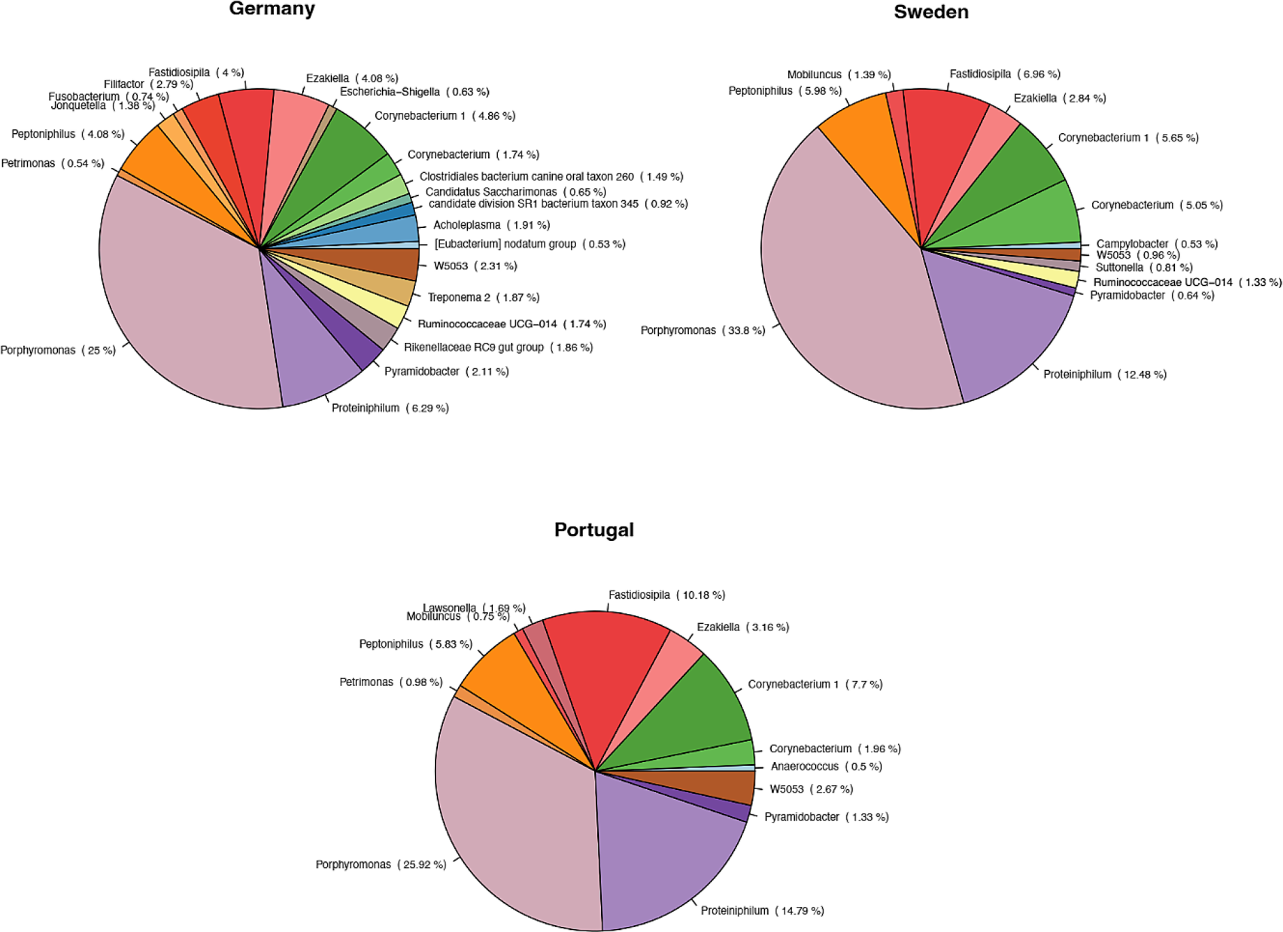
Mean relative abundance of Genera between the countries: Germany, Sweden, and Portugal

The community structure (beta diversity) in stallion semen, analysed using PERMANOVA tests with 10,000 permutations, showed significant differences (*p* < 0.05) between Germany and the other two countries in Bray-Curtis measurements (Figs. 4, 5 and 6). However, there was no difference (*p* > 0.05) between Sweden and Portugal. This was also observed with Unifrac and weighted-Unifrac measurements.

### Association with fertility

**Fig. 7:**
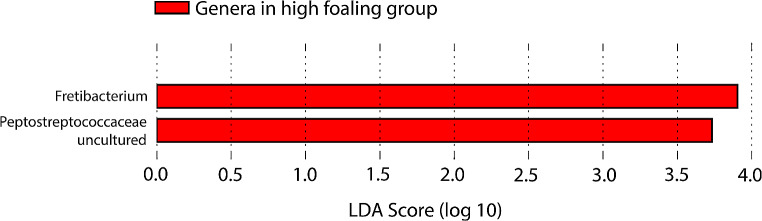
LDA score for *Fretibacterium* and uncultured genus from *Peptostreptococcaceae* family, was found abundant in the high fertility group. The length of the bar represents a log10 transformed LDA score. High foaling group = foaling rate > 60%

Two genera: *Fretibacterium* and a previously uncultured genus from *Peptostreptococcaceae* family appeared in the high fertility stallions to a greater extent than in the lower fertility stallions. The difference was significant with LEfSe analysis, with the effect size (LDA score) 3.9 (*p* = 0.033) and 3.73 (*p* = 0.0046), respectively (Fig. 7**)**.

### Temperature and precipitation

Mean annual temperatures and rainfall measured at meteorological stations near the studs in Germany, Sweden, and Portugal are shown in Table 3 (https://weather-and-climate.com/). The studs were in temperate regions with a similar maximum temperature in summer, although the number of months in which this temperature was reached was highest in Portugal and least in Sweden. The annual rainfall and number of rainy days was similar between Sweden and Germany, whereas Portugal had almost double the amount of rain but over fewer days. Humidity was similar in the three locations, although the local climate at the stud in Germany was slightly less humid than for the other two sites.

**Table 3:** Annual temperature, precipitation, rainy days and humidity at the three stud farms in Germany, Sweden, and Portugal (from https://weather-and-climate.com/, accessed date 11 August 2023)

	Relative Humidity (%)			Temperature (°C) min-max			Rainfall (mm)		
	Germany	Portugal	Sweden	Germany	Portugal	Sweden	Germany	Portugal	Sweden
Jan	80	81	82	(-2)-4	4–12	(-4)-1	44	125	31
Feb	78	80	80	(-1)-6	5–14	(-5)-1	37	115	26
Mar	66	76	75	1–9	6–16	(-2)-5	40	110	25
Apr	60	76	70	5–16	8–17	1–10	33	105	30
May	57	75	60	9–19	10–21	1–15	52	80	35
Jun	58	75	62	12–22	12–24	6–20	63	40	62
Jul	60	73	68	14–25	15–27	10–22	67	17	63
Aug	60	74	74	14–25	15–28	13–20	62	30	70
Sept	64	77	78	11–20	13–25	9–16	47	60	50
Oct	74	80	82	7–14	10–22	5–10	41	153	51
Nov	82	80	87	4–8	7–15	2–5	43	125	52
Dec	86	80	79	0–5	5–13	(-2)-3	160	148	163
Average RH, temp; total rainfall	69	77	75	6–14	9–19	4–11	160	148	163

## Discussion

The aim of this study was to determine whether there could be differences in the microbiome of semen from stallions kept in different geographical locations. The diversity of bacteria was indeed different between the three countries. The diversity was highest in Germany, then Portugal, and lowest in Sweden. However, the number of stallions from Sweden was much smaller than from the other two countries, due to convenience sampling. There may also have been differences between breeds, but the majority of the stallions in our study were warmbloods, with very few other breeds or types represented. To our knowledge, as yet there is no information about differences in the seminal microbiome among different equine breeds.

Three abundant phyla in our samples in Germany, Portugal, and Sweden, namely Bacteriodetes, Firmicutes, Actinobacteria, were similar to those in Spain (Quiñones-Pérez et al. 2021), in which four abundant phyla in semen samples from 12 stallion were Firmicutes, Bacteriodetes, Actinobacteria, and Proteobacteria. Despite the countries being geographically distant the abundant bacterial phyla were similar. Therefore, these phyla might represent the common bacteria residing in the reproductive tract of healthy stallions in European countries.

Eight abundant genera were *Peptoniphilus*, *Proteiniphilum*, *Fastidiosipila, Petrimonas Corynebacterium 1, Corynebacterium, Ezakiella*, and *W5053*. However, our results differed from a previous sequencing study in Sweden (Al-Kass et al. 2020) in which ten frequently seen genera were *Porphyromonas, Corynebacterium, Finegoldia, Peptoniphilus, Mobiluncus, Chondromyces, Suttonella, Treponema, Acinetobacter*, and *Campylobacter*. Even if the semen samples in the present study were collected from stallions at the same stud in Sweden as in a previous study and contained similar bacterial phyla, they differed at the genus level. The difference in bacterial genera might be due to the semen being collected in different years, or from different individuals, or at different times of the year.

In a previous study based on culturing bacteria from frozen-thawed semen in Portugal, the four genera isolated most frequently were *Enterococcus, Staphylococcus, Bacillus*, and *Corynebacterium* (Guimarães et al. 2015). In contrast, in our study using raw semen without antibiotics, the three most abundant genera identified by 16S sequencing were *Peptoniphilus*, *Proteiniphilum*, and *Fastidiosipila*.

The three most abundant genera in Germany from our study were *Peptoniphilus, Proteiniphilum*, and *Corynebacterium 1*; these were also different from a previous culture-based study in Germany. In the latter study, the four most frequently cultured genera were *Staphylococcus, Streptococcus, Corynebacterium*, and *Acinetobacter* (Pasing et al. 2013). However, the differences in bacteria might be due to the different methodologies used. Interestingly, the genus *Corynebacterium* was frequently found in stallion semen in both culture- and non-culture based studies.

The ASVs varied between Germany and Portugal, and between Germany and Sweden. A possible explanation for these differences is that many of the bacteria identified are environmental in origin. Therefore, climate and husbandry, as well as season, could be expected to influence the constituents of the microbiome of stallion semen (Al-Kass et al. 2020), at least at the level of genus and species. The stallions were kept in similar husbandry conditions e.g., they were housed individually and had access to paddocks for part of the day. The bedding material was not the same in the three locations, which might affect the bacterial load in the environment, as previously found in studies on cattle bedding (Hogan et al. 1989; Bradley et al. 2018). Pasing et al. (2013) investigated the microorganisms cultured from the genital mucosa and semen of stallions between February and August. They considered that an increase in the occurrence of *Staphylococcus vitulinus* from swabs of the penile sheath and urethral fossa during May might be due to environmental factors, or to changes in the quality of the bedding, or might be a reflection of an increased workload at the height of the breeding season among the staff caring for the animals. Interestingly, the present study was conducted at the same AI center as the one performed by Pasing et al. (2013),, and both studies may have included some of the same stallions.

The semen samples were collected in a different season in Sweden, which might have been expected to affect the microbial communities present. Most environmental bacteria are mesophilic (Nedwell, 1999) i.e. they do not grow below15°C; the temperature at the time of sampling would have been approximately 0–9 °C in Germany, somewhat lower than in Sweden (9–16 °C) or Portugal (7–19 °C for most of the samples). However, it was generally wetter in Portugal than in Sweden or Germany during the sampling period. Bacterial growth is fastest in conditions of high temperatures and high humidity (Qiu et al. 2022). Survival of bacteria such as Escherichia coli is affected by abiotic factors such as temperature, moisture and solar radiation (Jang et al. 2017), Therefore, one might have expected the conditions to be more favorable to bacterial growth in Portugal than in the other two locations, although this did not appear to be the case from our results. Semen samples from Germany, which experienced the coolest temperatures and an intermediate rainfall during the sampling period, had the greatest diversity of bacteria of the three groups. Therefore, factors other than climate appear to be important in determining the seminal microbiome.

Bacterial diversity might be connected to other factors not mentioned here. In a recent study on the microbial load in bull semen, the length of abstinence between collections (varying from one to three days) was found to influence seminal bacterial load, with the lowest levels in the ejaculates collected after one day´s abstinence (Cojkic et al. 2023). Unfortunately, information on the frequency of semen collection is lacking for the stallions in the present study. Although semen is collected regularly during the breeding season, either three times a week or every day, according to the requirements for semen from particular stallions, the samples from stallions in Portugal were collected when the stallions were presented for a breeding soundness examination rather than as part of a normal routine collection. The samples in Germany were obtained out of season at the start of the period of semen cryopreservation, whereas the end of the previous breeding season would have been August. However, the extra-gonadal sperm reserves had been depleted by semen collection in the week prior to the collections for this study. The period of abstinence in Portugal was likely to have been shorter since the samples were collected during the breeding season. However, a prolonged period of abstinence could explain the findings that the bacterial diversity was higher in the semen samples from Germany. As yet, no studies have been conducted to determine how many semen collections are needed to stabilize the bacterial populations in the reproductive tract.

Such findings could indicate a requirement to investigate the effects of bedding, husbandry, and management practices on microbial load in stallion semen, with a view to reducing antibiotic usage in semen extenders. Current practice is to use commercially available semen extenders containing broad spectrum antibiotics chosen by the manufacturer (Morrell and Wallgren 2014). Knowledge of the specific bacteria present in semen samples from particular locations might enable a more targeted approach to be used, choosing antibiotics that are effective for the bacteria prevalent at that location or finding alternatives (Morrell et al. 2022). Other factors that affect the microbial content of semen include hygiene practices but are not considered here. Such factors include whether or not to wash the penis of the stallion before semen collection, which is a common practice in some countries, or specifically at some studs (Kenney et al. 1975) but not in others (Bowen et al. 1982). In addition, discarding the pre-sperm fraction as in boar semen collection (Goldberg et al. 2013) could have an effect on the bacterial load. Furthermore, allowing the male to make several false mounts before collecting the semen, as in bull semen collection (Şahin et al., 2020) should, theoretically, help to flush bacteria out of the urethra before the ejaculate is collected. However, this practice is seldom used when collecting stallion semen. These factors are beyond the scope of the present study but should be included in extensive studies on controlling the bacterial content of semen. In any case, semen collection and processing should always be carried out with strict attention to hygiene (Althouse 2008).

The two bacteria *Fretibacterium* (Synergistetes phyla) and the unidentified genus from *Peptostreptococcaceae* family (Firmicutes phyla) were found abundant in the high fertility group compared to the low fertility group. Usually, an LDA score > 2 is considered as the threshold for a difference to be significant. *Fretibacterium* was previously isolated from the human oral cavity (Vartoukian et al. 2013). Numerous individuals from the *Peptostreptococcaceae* family are recognized as common residents of the gastrointestinal system. An unidentified genus of *Peptostreptococcaceae* was previously observed within domestic cats, constituting a substantial proportion of the fecal microbial population (Bermingham et al. 2018). Five stallions from Germany were found to have this bacterium in their semen samples, suggesting further investigation of the potential connection between cats and stallions at this stud.

There is a connection between the bacteria found in stallion semen and their negative effects on fertility (Varela et al. 2018), as well as their contribution to reduced sperm survival during storage (Ortega-Ferrusola et al. 2009). Therefore, it is important to know which bacteria are present in semen, to devise the best strategy of how to deal with them. It appears that some of the bacteria found in semen may he resistant to the antibiotics currently used in semen extenders (Guimarães et al. 2015). Knowledge of which bacteria are present in semen in a given country or area could help to identify if antibiotics are needed in semen extenders and which antibiotics would be the most suitable to use. The presence of previously unidentified bacteria in the semen samples is interesting, particularly as they are believed to be present in cats, although we do not know if there is a specific connection to cats at the stud farm in Germanys.

Our study population was restricted by necessity, since we could only obtain samples when the studs had access to stallions and had time to collect the semen when it was not needed for commercial AI. Therefore, the number of stallions was different in the different countries, and semen samples were collected at different times of year, with the samples from Sweden being obtained during the autumn. However, the result is striking; the seminal microbiome was different between Germany and Portugal, but was similar between Portugal and Sweden, suggesting that while climate may play a role in determining which organisms are likely to be present, other factors also influence the composition of this community. Further research is required to determine the optimum conditions for husbandry and semen collection routines to optimise the seminal microbiome.

In conclusion, the present results highlight the need for detailed knowledge of the seminal microbiome in stallions from particular geographic areas in order to provide targeted antibiotic usage in semen extenders. Bacteriodetes, Firmicutes, Actinobacteria, Synergistetes, and Proteobacteria were five abundant phyla in the semen samples from all three countries, similar to a previous study on stallion semen from Sweden. However, differences were observed at the genus level between the samples from the three countries, which may be due to differences in climate and husbandry, as well as to individual differences. An abundance of the genus *Fretibacterium*, as well as an unidentified genus from the *Peptostreptococcaceae* family, was observed in the high fertility stallions, which has not been documented previously.

## Data Availability

The datasets generated during and/or analysed during the current study are available from the corresponding author on reasonable request.
